# Underlying factors relating to acute myocardial infarction for coronary artery ectasia patients

**DOI:** 10.1097/MD.0000000000021983

**Published:** 2020-09-04

**Authors:** Qianqian Sheng, Huiqiang Zhao, Shanshan Wu, Ruifeng Liu

**Affiliations:** Department of Cardiology, Beijing Friendship Hospital Affiliated to Capital Medical University, Beijing, China.

**Keywords:** acute myocardial infarction, coronary artery ectasia, Markis classification

## Abstract

Coronary artery ectasia (CAE) patients were more prone to present with acute myocardial infarction (AMI), this case-control research aimed to explore the underlying factors relating AMI for them.

This study investigated a serial of 119 patients who underwent coronary angiography and were diagnosed as CAE between the years 2016 and 2017 at the Beijing Friendship Hospital; 32 of the patients developed AMI and 87 did not develop AMI. The possible factors relating to AMI, including disease history, cardiovascular risk factors, thrombotic condition, inflammation status, and coronary imaging characteristics, were comprehensively compared between CAE patients with and without AMI.

CAE patients with AMI had a lower antiplatelet rate, a higher blood low-density lipoprotein cholesterol level, increased neutrophil to lymphocyte (NL) ratio, higher Gensini score, and larger proportions of Markis type II. Logistic regression analysis also indicated that AMI history, lower antiplatelet rate, higher NL ratio, higher low-density lipoprotein cholesterol level and Gensini score, as well as Markis type II were associated with AMI in CAE patients.

AMI history, lower antiplatelet treatment rates, poor blood lipid control and higher coronary stenosis extent, higher inflammatory response, and Markis type II were closely related to the incidence of AMI in CAE patients.

## 1 Introduction

Coronary artery ectasia (CAE) is defined as dilatation of the coronary arteries to a diameter of more than 1.5 times its normal adjacent segment.^[1]^ Epidemiology data showed the incidence of CAE is about 1.4% to 12% in autopsies and during coronary angiography (CAG) or multidetector computed tomography angiography (MD-CTA),^[2–4]^ and more than 80% of CAE patients also had atherosclerotic stenosis. CAE can be divided into four types by the Markis classification method according ectasia extent.^[3]^ The main clinical manifestations were angina, acute myocardial infarction (AMI), arrhythmia, and sudden death, while some patients had no obvious symptoms.^[2]^ CAE was more likely a thrombotic disease, as Swaye PS reported that AMI prevalence in CAE populations was 52.9%, which was higher than in patients with coronary heart disease (CHD) and in other non-CAE patients.^[5]^ According to our limited clinical practices, a portion of but not all CAE patients were more prone to present with AMI^[6]^ and even recurring AMI. The exact underlying mechanisms as to why some patients with CAE tended to develop AMI remain unclear. Thus, it was necessary to distinguish CAE patients with high AMI risk in the early screening stage in order to prevent AMI events from interfering with the controllable factors. This study aimed to explore the underlying factors relating to AMI in CAE patients by systematically evaluating the disease histories, cardiovascular risk factors, thrombotic systems, inflammation conditions, and coronary imaging characteristics in CAE populations. These results would be useful for clinical practice and future research in this field.

## 2 Patients and method

### 2.1 Patient population

This research was a case-control study. A total of 5012 subjects who underwent CAG at the cardiac catheterization center of Beijing Friendship Hospital between January 2016 and December 2017 were screened for participation in the study; among them, 119 (2.37%) were eligible according to inclusion and exclusion criteria as following and identified as having CAE. These 119 patients were then divided into 2 groups: 1 group of 32 CAE patients with AMI (CAE+AMI group) and another group of 87 patients without AMI (CAE group).

The baseline data, key laboratory tests, and imaging character were compared and analyzed comprehensively between the above 2 groups. Then the effect of ectasia extent on AMI prevalence was investigated by comparing the different Markis types. Furthermore, logistic analysis was applied by setting AMI as the dependent factor and other items as independent parameters.

This study was approved by the ethics committee of Beijing Friendship Hospital Affiliated to Capital Medical University and was in accord with the Declaration of Helsinki. Informed consent was obtained from all study participants.

### 2.2 Inclusion criteria

A diameter of the ectatic segment that was 1.5 times greater than that of the adjacent normal segment was defined as CAE.^[4]^ According to current consensus,^[7]^ AMI was defined by an elevated cardiac biomarker and at least 1 of the following:

(1) symptoms relating to ischemia,
(2) changes on an electrocardiogram, such as ST segment changes, new left bundle branch block, or Q waves,
(3) changes in the motion of the heart wall on imaging, and/or
(4) discovery of a thrombus on angiogram or at autopsy.

### 2.3 Exclusion criteria

Exclusion criteria were cardiomyopathy, myocarditis, valvular heart disease, chronic heart failure, aneurysm in other vessels, collagen tissue diseases, vasculitis, syphilis, explicit diagnosis of pulmonary embolism and aortic dissection, severe liver and kidney dysfunction, hemagglutination disorders, pregnancy, active bleeding, malignant neoplasm, infectious diseases, previous history of infection (<3 months), other inflammatory diseases, and poor compliance.

### 2.4 Basic clinical characteristics

The medical records and angiography databases were intact and detailed. Most of the data were obtained from the medical record system and the radiography database, including demographics (such as age and sex), history of past diseases (such as hypertension, diabetes, coronary heart disease, etc), family history, personal history (such as smoking and drinking), and history of past drug use. Body mass index was calculated by dividing weight in kilograms by height in meters squared (kg/m^2^). The left ventricular ejection fraction (LVEF) and the peak flow rate ratio (E/A) in early and late diastolic phase were evaluated by transthoracic echocardiography (Philips EPIQ 7C with a S5-1 probe). The LVEF for all subjects was first measured by M-mode ultrasound method. Then if the patient was with enlarged heart, or segmental abnormal wall motion, or ventricular aneurysms, the biplane Simpson method would be applied to introduce a new LVEF value as the final measurement.

### 2.5 Biochemical indicators

For all subjects, blood samples were analyzed by the Clinical Laboratory of Beijing Friendship Hospital. For most laboratory measurements, the vein blood sample was extracted in the morning after the patient was admitted to the hospital. The main items included were total cholesterol; triglycerides; high density lipoprotein cholesterol; low density lipoprotein cholesterol (LDL-c); leukocyte count; neutrophils; lymphocytes; monocytes; neutrophil to lymphocyte (NL) ratio; total bilirubin and direct bilirubin, which functioned as anti-oxidant factors to some degree;^[8]^ high sensitivity C-reactive protein (hs-CRP); and erythrocyte sedimentation rate. The thrombelastography was analyzed after the patient was admitted to the hospital to ensure systematic evaluation of the thrombotic condition.

### 2.6 Coronary artery imaging features

Coronary angiograms were performed via a radical artery or femoral artery approach, without the use of adenosine or calcium channel blockers. According to the Markis classification method, the CAE could be classified into 4 groups based on the extent of coronary involvement: type I, diffuse ectasia of 2 or 3 vessels; type II, diffuse disease in 1 vessel and localized disease in another vessel; type III, diffuse ectasia of 1 vessel only; and type IV, localized or segmental ectasia.^[3]^ The thrombolysis in myocardial infarction (TIMI) frame count (TFC)^[9]^ of left anterior descending coronary artery, left circumflex coronary artery, and right coronary artery were intuitively determined by 2 experienced cardiac interventionist experts according to strict anatomical signs and imaging characteristics. The Gensini scores^[10]^ were calculated to evaluate the coronary stenosis extent at the same time.

### 2.7 Statistical analysis

The Statistical Package for Social Sciences software (SPSS 25.0, IBM SPSS Statistics, Armonk, New York) was used for the analyses. All data were initially analyzed using the Kolmogorov-Smirnov test to assess for normality. Continuous data are presented as mean ± SD when normally distributed and median with interquartile range (IQR) when non-Gaussian in distribution. Unpaired *t-*tests and Mann-Whitney-*U* rank sum tests were used for bivariate analyses of normally and non-normally distributed continuous data, respectively. Then, the logistic analysis was applied by setting with forward method and threshold of 0.05; *P* value <.05 was considered significant.

## 3 Results

### 3.1 Baseline characteristics for enrolled subjects

According the medical database from January 2016 to December 2017 in our hospital, CAE prevalence was 2.37% (119/5012) in the subjects who underwent CAG evaluation. There were 105 CAE patients with CHD (105/119 = 88.24%) and 29 CAE + AMI patients with CHD (29/32 = 90.63%), while the CHD prevalence was 92.03% (4503/4893) in non-CAE patients. The AMI prevalence rate in CAE patients was 26.89% (32/119), which was higher than in non-CAE population who underwent coronary angiograms 19.15% (937/4893).

The baseline characteristics of the 2 groups are shown in Table 1. The 2 groups were balanced with regard to age, sex, CHD history, hypertension, blood pressure, diabetes, smoking, alcohol, and body mass index. CAE + AMI patients had higher AMI history rates (28.13% versus 8.05%, *P* = .012). The LVEF were 61.61% ± 6.45% for CAE + AMI patients and 66.07% ± 6.98% for CAE group (*P* = .003).

**Table 1:**
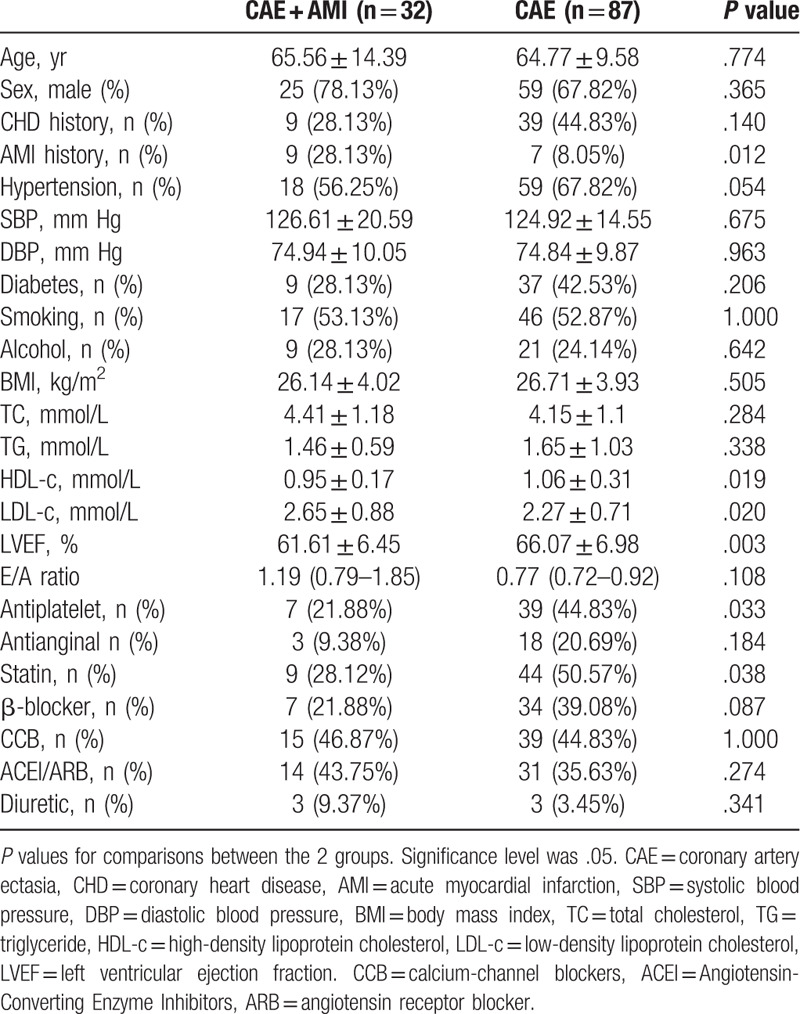
Baseline characteristics for enrolled subjects.

There was no significant difference between the CAE + AMI group and CAE group regarding total cholesterol and triglyceride. High-density lipoprotein cholesterol was lower and LDL-c was higher, with significant statistical differences (0.95 ± 0.17 vs 1.06 ± 0.31, *P* = .019, 2.65 ± 0.88 vs 2.27 ± 0.71, *P* = .020) in CAE + AMI group compared to the CAE group (Table 1).

At the same time, the antiplatelet treatment rate, and the statin treatment rate before admission was lower in CAE + AMI group (21.88% vs. 44.83%, *P* = .033; 28.12% vs 50.57%, *P* = .038) while most of medications before admission were balanced between the 2 groups (Table 1).

### 3.2 Thrombotic system evaluation and inflammation indicators

The thrombotic system, including platelet functions, coagulation system, and fibrinolytic system could be evaluated by thrombelastogram in Table 2. There was no significant difference in thrombelastogram between the 2 groups (*P* > .05).

**Table 2:**
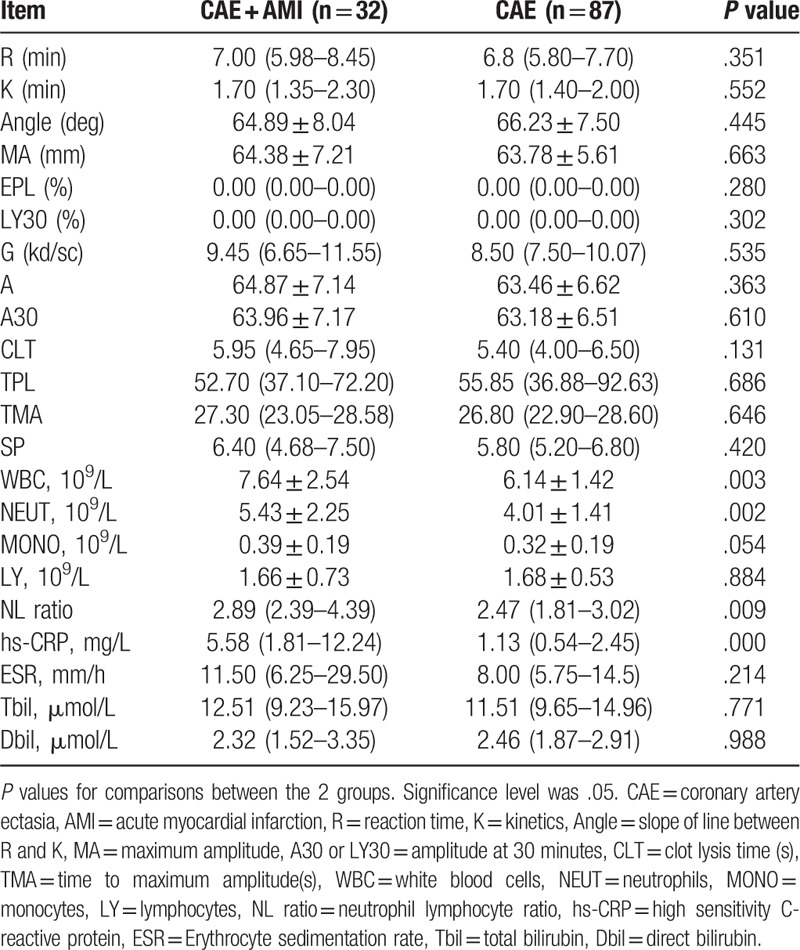
The thrombelastogram analysis and inflammation indicators.

The white blood cell count (7.64 ± 2.54 vs 6.14 ± 1.42, *P* = .003), neutrophil count (5.43 ± 2.25 vs 4.01 ± 1.41, *P* = .002), NL ratio (2.89 [2.39–4.39] vs 2.47 [1.81–3.02], *P* = .009) and hs-CRP (5.58 [1.81–12.24 vs. 1.13 [0.54–2.45], *P*= .000) in the CAE + AMI group were significantly higher than those in the CAE group (Table 2).

### 3.3 Coronary imaging characteristics

This study showed in Table 3 that in general Markis type III was the most common type in CAE patients. Markis type II was higher with AMI (34.38% vs 14.94%, *P* = .037) in the CAE + AMI group than in the CAE group, while Markis type IV was lower with AMI (9.38% vs 31.03%, *P* = .017). The ectasia sites were more common in right coronary artery with an incidence of 66.39% (79/119). The incidence of infarction within ectasia part was 59.38%.

**Table 3:**
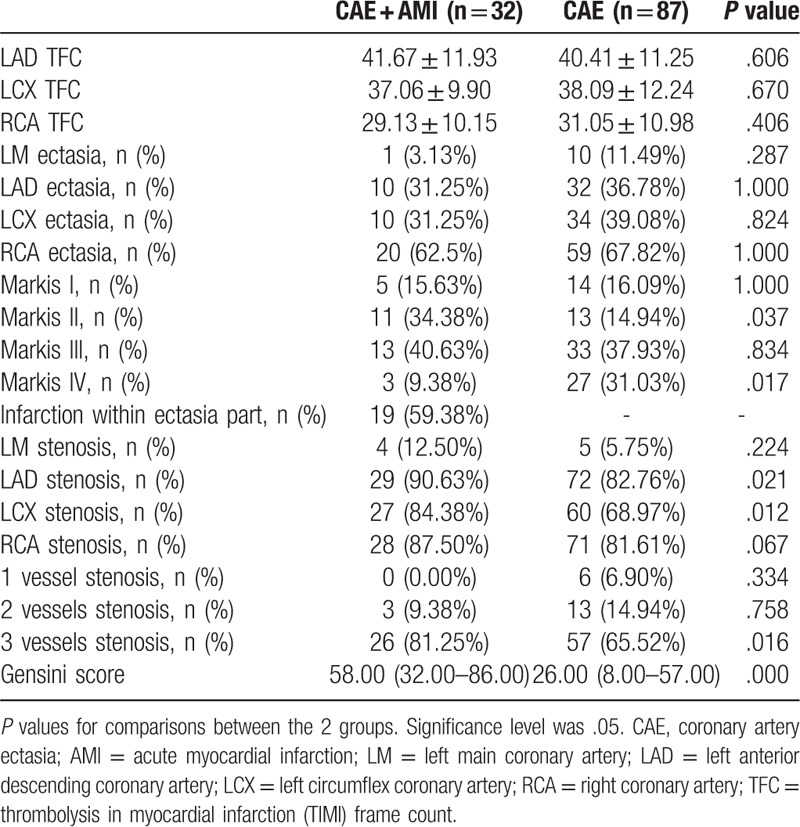
Coronary imaging characteristics for enrolled subjects.

The Gensini score was significantly higher in patients with AMI compared to patients without AMI (58.00 [32.00–86.00] vs 26.00 [8.00–57.00], *P* = .000). The CAE + AMI group had higher rates of left anterior descending coronary artery stenosis and left circumflex coronary artery stenosis (90.63% vs 82.76%, *P* = .021; 84.38% vs 68.97%, *P* = .012) and 3-vessels stenosis (81.25% vs 65.52%, *P* = .016) than the CAE group (Table 3).

### 3.4 Subgroup analysis for CAE patients

According to the Markis classification method,^[3]^ the 119 patients were divided into four groups. The results showed the AMI prevalence for Markis II CAE patients was 45.83%, which was higher than that of the other 3 groups. And another result is the corrected thrombolysis in myocardial infarction (TIMI) frame count of Markis I patients was higher than other 3 groups. (Table 4).

**Table 4:**
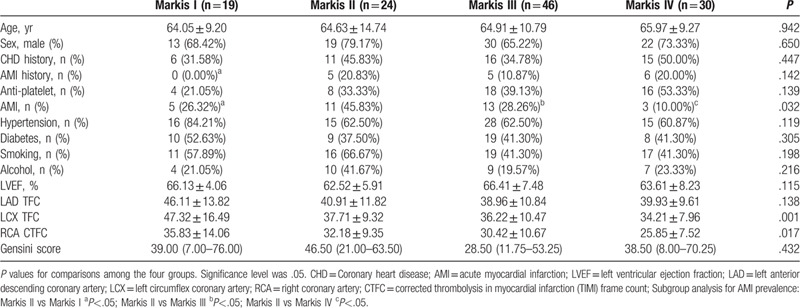
The comparisons among CAE subgroups classified by Markis method.

### 3.5 Logistic regression analysis for AMI in CAE patients

Logistic regression analysis was applied in Table 5 to explore the factors relating to AMI for CAE patients. In this setting, AMI was the dependent variable, and other factors that may relate to AMI were arranged as independent variables. This study used the forward method and set the corresponding p value at less than 0.05. The logistic regression analysis revealed that AMI history, lower antiplatelet rate before admission, higher NL ratio, higher blood LDL-c level (means poor lipid control), Gensini score (higher Gensini score means more extent in stenosis), as well as Markis type II were associated with AMI in CAE patients.

**Table 5:**
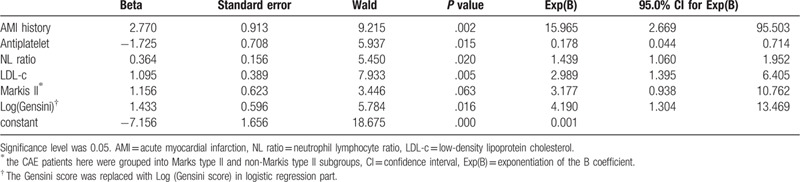
Logistic regression for possible items relating to AMI for CAE patients.

## 4 Discussion

As known more than 80% of CAE were combined with CHD stenosis,^[2–4]^ and in CAE patients including those with CHD and without CHD, AMI and sudden death rate were higher than that of pure CHD patients,^[10–13]^ thromboembolism might be the main reason.^[14,15]^ The expansion site was often the site of thrombus formation (culprit vessels).^[12,16]^ If CAE patients have coronary artery thrombotic events, the reperfusion treatment success rate is lower than in non-CAE patients, the large thrombus also increased the no reflow phenomenon and distal embolization and other incidence of adverse events during percutaneous coronary intervention,^[2,14]^ and after percutaneous coronary intervention it was also prone to have stent thrombosis.^[17]^ Thus CAE is a kind of thrombotic disease in some degree and it is necessary to find out the underlying reasons especially the controllable factors relating to AMI in CAE patients. In this research, by comparisons between the AMI + CAE group and CAE group, by comparison among the 4 Markis subgroups, and by logistic regression analysis in which AMI was dependent variable, it was founded possible factors relating to AMI in CAE patients were:

(1) AMI history, lower antiplatelet rate,
(2) higher NL ratio and other inflammation indicators,
(3) higher Gensini score, and higher LDL-c level,
(4) as well as Markis type II CAE. Those risk factors relating to AMI for CAE founded in this research were very similar to those for CHD.

In fact, most of CAE patients did have obvious atherosclerosis manifestations in coronary and other vessels: the coronary calcification score,^[18,19]^ the carotid artery intima-media thickness^[20–23]^ and pulse wave velocity^[24]^ were all significantly increased in CAE patients. As to pathological characteristics in coronary:

(1) there was significant atherosclerotic change in intimal and media of coronary artery;
(2) inflammatory cells (neutrophils, lymphocytes, plasma cells, eosinophils and mononuclear cell) were infiltrated in the vessel wall.^[3,25]^

The pathological features of CAE in general were similar to CHD except the damage of smooth muscle and elastic fiber layer on the middle coronary wall which might be due to proteolysis.^[25–27]^ And also similar to CHD, CAE patients were in a systemic inflammatory state with higher level of hsCRP and NL ratio.^[28–31]^ It was suggested CAE and CHD might have a common pathological process with a viewpoint that CAE was a variation from CHD.^[32]^ Theoretically some novel candidate indicators for atherosclerosis, inflammation and prognosis in CHD and AMI patients such as pentraxin 3^[33]^ and osteoprotegerin^[34]^ might be also important for CAE patients. Those AMI relating factors for CAE patients founded in this study might have important clinical significance.

First, CAE patients with high AMI risk could be distinguished if they presented with 1 or more following characters: AMI history, lower antiplatelet treatment rates, poor blood lipid control and higher coronary stenosis extent, higher inflammatory response, and Markis type II. There might be unknown intrinsic factors relating to thrombotic event for CAE patients, thus the patients with AMI history and lower rate of antiplatelet were more likely to undertake AMI, a special CAE case in our hospital had experienced AMI four times within 1 year with dual antiplatelet treatment. The severer inflammation status and atherosclerosis changes in coronary artery might contribute to this kind of higher AMI risk in some degree. The inflammation status and poorer-controlled blood lipid were promotive factors for atherosclerotic plaques formation indicated by higher Gensini scores. As to why Markis type II patients was with highest AMI prevalence, TA Zografos also observed AMI was more frequent in patients with diffuse ectasia,^[35]^ but the relationship between AMI risk and Markis type had not been clearly addressed until present.

Secondly, this research indicated the possible solutions for preventing AMI in CAE patients might include antiplatelet treatment, anti-atherosclerosis treatment including lipid-lowering treatment, and so on. Correspondingly in practices, most proposals for CAE were based the treatment of CHD currently, and anti-thrombotic agent and statin were the main treatments. For antiplatelet treatment, there is no consensus whether to select a single antiplatelet agent, dual antiplatelet therapy or the combination of antiplatelet agent and anticoagulant therapy. Some research notes the larger coronary aneurysms require aggressive treatment through a combination of antiplatelet and anticoagulant therapy.^[36]^ For the anti-atherosclerosis treatment, in theory the application of statin was essential for CAE patients not only by lipid-lowering effect but also by anti-inflammation as for CHD patients, but the lipid-lowering goal, the anti-thrombosis effect of statin were unknown as so far. More clinical trails were needed to evaluate the efficacy of anti-thrombotic agent and statin for CAE patients and to observe the long-term prognosis.

## 5 Conclusion

The possible factors relating to AMI for CAE patients were higher AMI history, lower antiplatelet treatment rate, poor blood lipid control and higher coronary stenosis extent, higher inflammatory status, and Markis type II. More attention should be paid to these factors in order to decrease the AMI rate for CAE patients.

### 5.1 Limitations

This research has some deficiencies and limitations that need to be further improved:

(1) it was a small sample size and single centre study because it was not easy to enroll enough subjects due to the low prevalence of CAE and it was usually diagnosed by special methods such as CAG and coronary MD-CTA but not by other easier and more popularized methods;
(2) this study did not conduct a follow-up observation, and the short and long-term effects of those underlying factors especially the treatments of anti-platelet agent and statin needed to be further evaluated.
(3) It was a clinical research by analysis of the data from routine medical practices, other molecules and biological mechanisms which might be also related with AMI for CAE patients were not considered and evaluated by this research.

## Acknowledgments

This study was supported by the National Natural Science Foundation of China (Grant No. 81600276).

## Author contributions

**Conceptualization:** Qianqian Sheng and Ruifeng Liu

**Formal analysis:** Qianqian Sheng and Ruifeng Liu

**Investigation:** all of authors

**Methodology:** Shanshan Wu

**Project administration:** Qianqian Sheng and Ruifeng Liu Qianqian Sheng and Ruifeng Liu conceived the study and its design;

**Resources:** Qianqian Sheng and Ruifeng Liu

**Software:** Qianqian Sheng and Ruifeng Liu

**Validation:** all of authors

**Writing – original draft:** Qianqian Sheng

**Writing – review & editing:** Ruifeng Liu and Qianqian Sheng
